# Percutaneous RF ablation versus surgical RF assisted nodulectomy in early stage HCC; our experience in elderly patients

**DOI:** 10.1186/1471-2482-13-S1-A46

**Published:** 2013-09-16

**Authors:** Alfonso Terrone, Alberto Oldani, Clemente De Rosa, Manuela Monni, Marcello Garavoglia

**Affiliations:** 1 Department of Surgery University of Eastern Piedmont “Amedeo Avogadro, Hospital “Maggiore della Carità” Novara, Italy

## Introduction

Surgical resection usually represents the treatment of choice for solitary hepatocellular carcinoma (HCC) in cirrhotic patients, with well preserved liver function; local ablative strategies are the best treatment option for patients with small tumors who are not candidates for surgical resection or liver transplantation [1].

The incidence of HCC increases whit age, reaching its highest prevalence among patients with more than 65 years [2].

The current literature shows a better outcome in patients with tumors smaller than 3 cm especially after percutaneous RFA [3].

Several studies showed that percutaneous radiofrequency ablation (RFA) has similar efficacy to surgical nodulectomy in the treatment of early-stage HCC, and is associated with lower complication rates and costs than resection [4].

The aim of this study is to compare the effectiveness of these treatments, in terms of morbidity, overall survival, tumor recurrence and cause of death.

## Methods

Between January 2006 and January 2012 we observed 176 patients affected by HCC, 84 underwent curative treatment.

The 40 patients presenting single HCC nodes smaller than 3 cm in diameter have been treated whit radiofrequency-assisted surgical nodulectomy or with percutaneous radiofrequency.

Of these 40 patients , 23 patients were age greater than or equal to 75 years.

We divided these patients in two homogeneous groups: Group A (11 patients) treated with RF assisted surgical nodulectomy; Group B (12 patients) treated with percutaneous RFA.

The two groups result homogeneous by age, degree of liver function and tumor size.

In all cases HCC was diagnosed and staged whit classical techniques such as basic and contrast enhancement ultrasound imaging, computed tomography (CT), and magnetic resonance imaging (MRI).

All surgical procedures obtained radical resection of the tumor.

## Results

Postoperative and post ablation mortality was 0.

Morbidity rates were 9.09% in Group A (1 case of peritoneal bleeding needing re operation) and 8.33% in Group B (1 case of liver abscess).

Overall mortality was 36.36% in Group A (1 patient died for end stage liver cirrhosis and 3 patients for metastatic disease 33.33% in Group B (2 patients died for local tumor recurrence and 2 patients for cardiovascular events).

No statistically significative difference were observed in terms of morbidity, overall and disease free survival between the two groups.

## Conclusions

Many experience in literature show that RF and surgical resection can be used in curative treatment of HCC with good results; the less invasiveness in the main advantage of percutaneous RF application patients with compromised liver function and high stage cirrhosis: performing nodulectomy with RF-assisted liver section reduces blood loss and the need of transfusion [5].

The choice of clinical approach must be discussed between surgeons and radiologist, taking into consideration the operator’s experience and also the patient’s opinion.

Despite the relatively small number of patients observed our study confirm that RF and surgical resection can be either used in HCC treatment even in elderly patients, with no differences in terms of complication rate, overall and disease free-survival rates.

Surgical approach seems to prevent local recurrence.
